# DNA demethylation caused by 5-Aza-2′-deoxycytidine induces mitotic alterations and aneuploidy

**DOI:** 10.18632/oncotarget.6897

**Published:** 2016-01-12

**Authors:** Giuseppe Costa, Viviana Barra, Laura Lentini, Danilo Cilluffo, Aldo Di Leonardo

**Affiliations:** ^1^ Dipartimento di Scienze e Tecnologie Biologiche, Chimiche e Farmaceutiche (STEBICEF), Università degli Studi di Palermo, Palermo, Italy; ^2^ Centro di OncoBiologia Sperimentale (COBS), Palermo, Italy

**Keywords:** DNA demethylation, 5-aza-2′-deoxycytidine (DAC), aneuploidy, chromosome methylation pattern, Chromosome Section

## Abstract

Aneuploidy, the unbalanced number of chromosomes in a cell, is considered a prevalent form of genetic instability and is largely acknowledged as a condition implicated in tumorigenesis. Epigenetic alterations like DNA hypomethylation have been correlated with cancer initiation/progression. Furthermore, a growing body of evidence suggests the involvement of epigenome-wide disruption as a cause of global DNA hypomethylation in aneuploidy generation.

Here, we report that the DNA hypomethylating drug 5-aza-2′-deoxycytidine (DAC), affects the correct ploidy of nearly diploid HCT-116 human cells by altering the methylation pattern of the chromosomes. Specifically, we show that a DAC-induced reduction of 5-Methyl Cytosine at the pericentromeric region of chromosomes correlates with aneuploidy and mitotic defects.

Our results suggest that DNA hypomethylation leads to aneuploidy by altering the DNA methylation landscape at the centromere that is necessary to ensure proper chromosomes segregation by recruiting the proteins necessary to build up a functional kinetochore.

## INTRODUCTION

The majority of human tumours show a form of genome instability called chromosomal instability (CIN) that refers to the high rate of numerical and structural chromosome aberrations found in cancer cells. Numerical CIN is characterised by gain and loss of whole chromosomes resulting in aneuploidy, a harmful condition for the viability of cells and organisms. At the molecular level, aneuploidy arises by several mechanism(s) including mutations in genes encoding mitotic regulators [1, 2], tumour suppressors or controlling centrosome numbers [3-7], altered expression of mitotic checkpoint proteins, defects in chromatid cohesion and in kinetochore-microtubule attachment [6-10]. Furthermore, epigenetic alterations such as DNA hypomethylation are considered as a cause of aneuploidy [11, 12]. Global genome hypomethylation has been described in breast, ovarian, cervical and brain tumours and it has been reported that severe hypomethylation correlates with increased malignancy [13]. However, little is known about the mechanism(s) by which most of human cancers become aneuploid following DNA hypomethylation. DNA hypomethylation can result in different phenotypic effects depending on the cell genotype. Previously, we reported that DNA Methyl-Transferase 1 (DNMT1) depletion caused cell cycle arrest in IMR90 cells and aneuploidy in HCT-116 colon cancer cells missing p14ARF function in association with global DNA hypomethylation [14]. Loss of DNMTs function leads to global genome hypomethylation and to chromosomal instability in mouse models [15] and human ICF syndrome [16, 17]. Specifically, cells from patients with ICF syndrome exhibit hypomethylation of pericentromeric regions associated with the formation of micronuclei [18].

Two different hypotheses, not mutually exclusive, have been formulated to explain the correlation between DNA hypomethylation and aneuploidy. DNA hypomethylation could alter the expression of genes coding for specific mitotic checkpoint proteins. Alternatively modifications of chromosomal DNA methylation pattern could also interfere with the correct structure of chromosomes [12]. In this regard, it has been reported that an epigenetic, sequence-independent mechanism underlies the formation of the centromere [19, 20]. Indeed, the epigenetic environment onto and around the centromere is very important to ensure proper chromosome segregation. In all species studied, the centromere is defined by a specific chromatin domain containing a H3 histone variant (CENP-A), flanked by a large pericentromeric heterochromatin domain CENP-A free and enriched in highly repetitive α-satellite sequences. Although repetitive DNA sequences are not essential for centromere formation, they provide the necessary environment to assemble the centromere [21 22] and the disruption of pericentromeric heterochromatin conformation leads to chromosome segregation defects, generally associated with the loss of cohesins into and around this domain [20, 23, 24].

The decrease of pericentromeric methylation in human lymphocytes treated with the 5-Azacytidine (AZA) inducing loss of DNA methylation was associated with missegregation of chromosomes 1 and 16 [25]. Given that these two chromosomes have highly methylated satellite 2 regions this observation suggests a relationship between loss of DNA methylation and chromosomes loss.

To investigate the role of global DNA hypomethylation on chromosome structure and segregation and to clarify the relationship between DNA hypomethylation and generation of aneuploidy, we treated HCT-116 cells, a nearly diploid human cell line with 5-aza-2′-deoxycytidine (DAC, also known as decitabine), to induce DNA hypomethylation [26]. After cellular uptake and phosphorylations, the cytidine analogue AZA is incorporated into both DNA and RNA with effects on the processing of tRNA and thereby interfering with protein translation. On the contrary, DAC is incorporated into DNA and causes more efficient inhibition of DNA methyltransferases [27]. DAC acts through the formation of covalent DNMT1-DAC-DNA adducts leading to a decrease in DNMT1 levels and CpG dinucleotides hypomethylation [28]. This in turn causes changes in the genome methylation pattern to be transmitted to daughter cells.

Here, we report that the DNA hypomethylating drug 5-aza-2′-deoxycytidine (DAC), affects the correct ploidy of nearly diploid HCT-116 human cells by altering the methylation pattern of the chromosomes. Specifically, we show that a DAC-induced reduction of 5-Methyl Cytosine at the pericentromeric region of chromosomes correlates with aneuploidy and mitotic defects

## RESULTS

### Low doses of DAC induce DNA hypomethylation and aneuploidy

**Figure 1:**
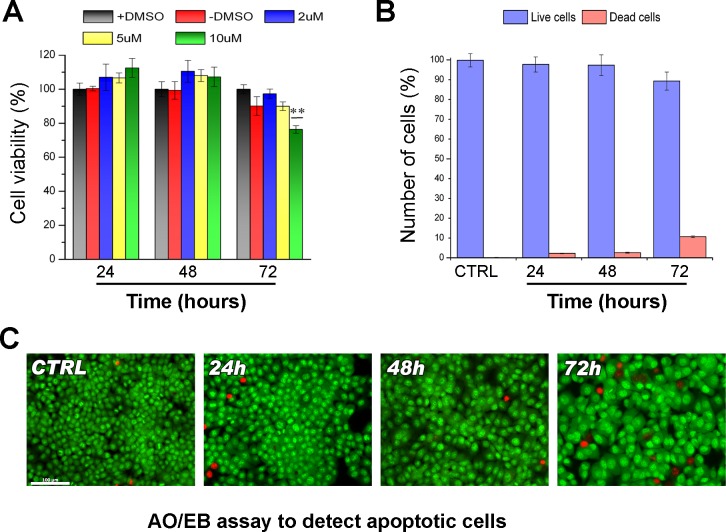
Effects of DAC on viability of HCT-116 cells **A**. Bar graph showing the viability of HCT-116 treated with different DAC concentrations (2, 5 and 10 μM) at different times (24h, 48h and 72h), A treatment with only vehicle (+DMSO) and a no-treated sample (−DMSO) are included as controls. The statistics was performed applying the ANOVA test with Bonferroni correction ** (P<0.01; n=3). **B**. Histogram showing the values (%) of living and dead cells stained with Acridine Orange/Etidium Bromide (AO/EB) after DAC treatment at different concentrations and times (24, 48 and 72h). **C**. Images of the different cell populations after AO/EB staining observed with fluorescence microscopy. Living cells appear green, dead cells red.

**Figure 2:**
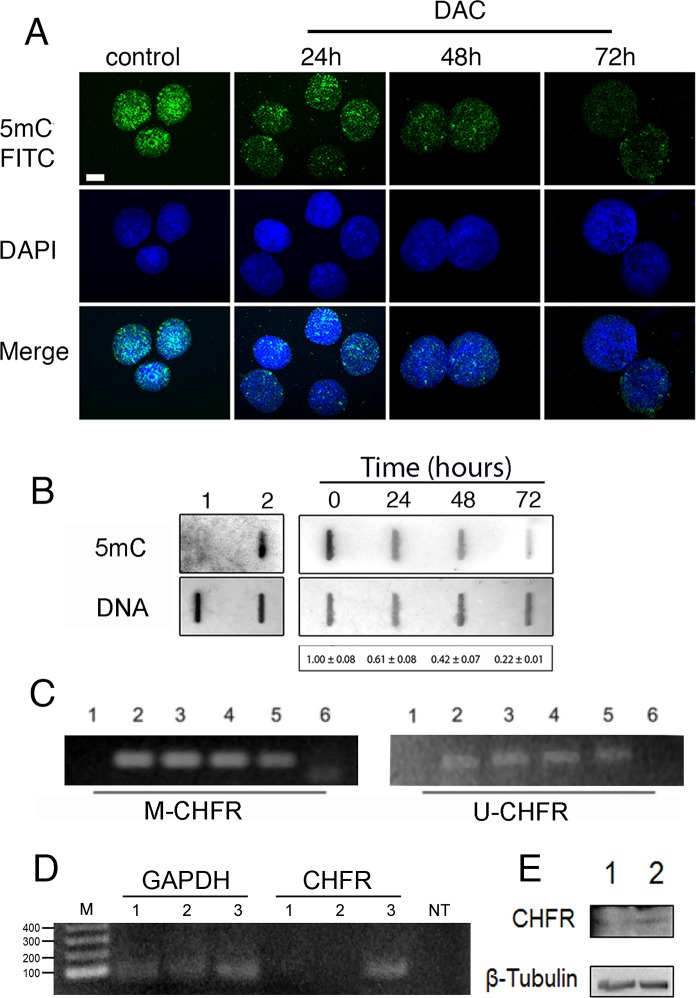
Low doses of DAC induce DNA de-methylation A, immunofluorescence microscopy was performed to detect 5-mC signals in HCT-116 nuclei after treatment with 5 μM DAC for 24-, 48- and 72 h. **A**. DMSO treatment was included as control. **B**. slot-blot analysis detecting 5-mC in DAC treated HCT-116 cells (0h-72h, top right panel). The amount of spotted DNA was detected by methylene blue staining (bottom right panel). The values vs control ± SEM are indicated under the panel. The left panel shows the non-methylated DNA of Escherichia coli ET12567/pUZ8002 used as a negative control (1), and the methylated DNA of Escherichia coli Yale BW25113 used as a positive control (2). Data are from three independent experiments. **C**. Methylation Specific PCR shows the methylation status of the CHFR gene promoter in HCT-116 cells treated with 1, 2 and 5μM DAC for 72h (lanes 3, 4, 5, respectively). The “M-CHFR” and the “U-CHFR” panels show amplification products obtained by using primers for methylated and unmethylated CHFR promoter, respectively. SW480 (1) and HCT-116 cells (2) were used as negative and positive control in M-CHFR panel, the opposite HCT-116 (1) and SW480 (2) in the U-CHFR panel. Lane 6 is no template. **D**. RT-PCR showing expression levels of CHFR in HCT116 untreated (lane 1) and treated with DAC (5μM) for 24h (lane 2) or 48h (lane 3). GAPDH was used as an internal control. **E**. Western Blot showing the presence of CHFR protein in HCT116 cells after 48 hours of treatment with DAC 5μM (lane 2) in respect to HCT116 left untreated (lane 1). β-tubulin was used as a loading control.

DNA methylation occurs onto cytosine located in the so-called CpG islands thanks to DNA methyl-transferases. DNA methylation strongly influences chromatin structure and generally leads to a higher degree of compaction thus repressing gene expression. In order to study the effects of DNA hypomethylation on chromosome dynamics, we first assessed at which concentration DAC was able to induce DNA hypomethylation without affecting cell viability and cell proliferation. HCT-116 cells were treated with DAC at different doses and the effects of exposure were evaluated over a period of 72 hours. Cells treated with 2 μM and 5μM of DAC did not show reduction of cell viability (Figure 1A). In addition the acridine orange assay showed that HCT116 cells after DAC treatment did not undergo apoptosis or necrosis (Figure 1B). On the contrary, DAC at a concentration of 10 μM severely impaired cell viability (Figure 1A). Next, we assessed the level of 5-methylcytosine (5mC) in HCT-116 cells after the DAC treatment. Fluorescence microscopy and Slot-blot analysis showed a progressive and intensive reduction of the 5-mC signal throughout 72 hours of treatment with a decrease of 50%, 60% and 80% after 24, 48 and 72 hours, respectively (Figure 2A, 2B; Figure S1). This result is in accordance with the DAC mechanism affecting DNA methylation level via CpG dinucleotides hypomethylation [28] that in turn causes changes in the genome methylation pattern of daughter cells. To determine if loss of methylation upon DAC treatment occurred also at specific hypermethylated promoter we evaluated the promoter methylation status of the CHFR gene that is hypermethylated in HCT-116 cells [29]. The methylation status of the CHFR promoter was assessed by Methylation Specific PCR (MSP) in HCT-116 cells treated with DAC (1, 2, 5μM) for 72 hours. DAC treatment induced loss of DNA methylation of the CHFR gene promoter as revealed by the presence of an amplicon obtained with specific primers annealing with the unmethylated DNA (Figure 2C). The methylation loss of CHFR promoter was confirmed by the re-expression of CHFR assessed by RT-PCR and Western Blot (Figure 2D, 2E).

**Figure 3:**
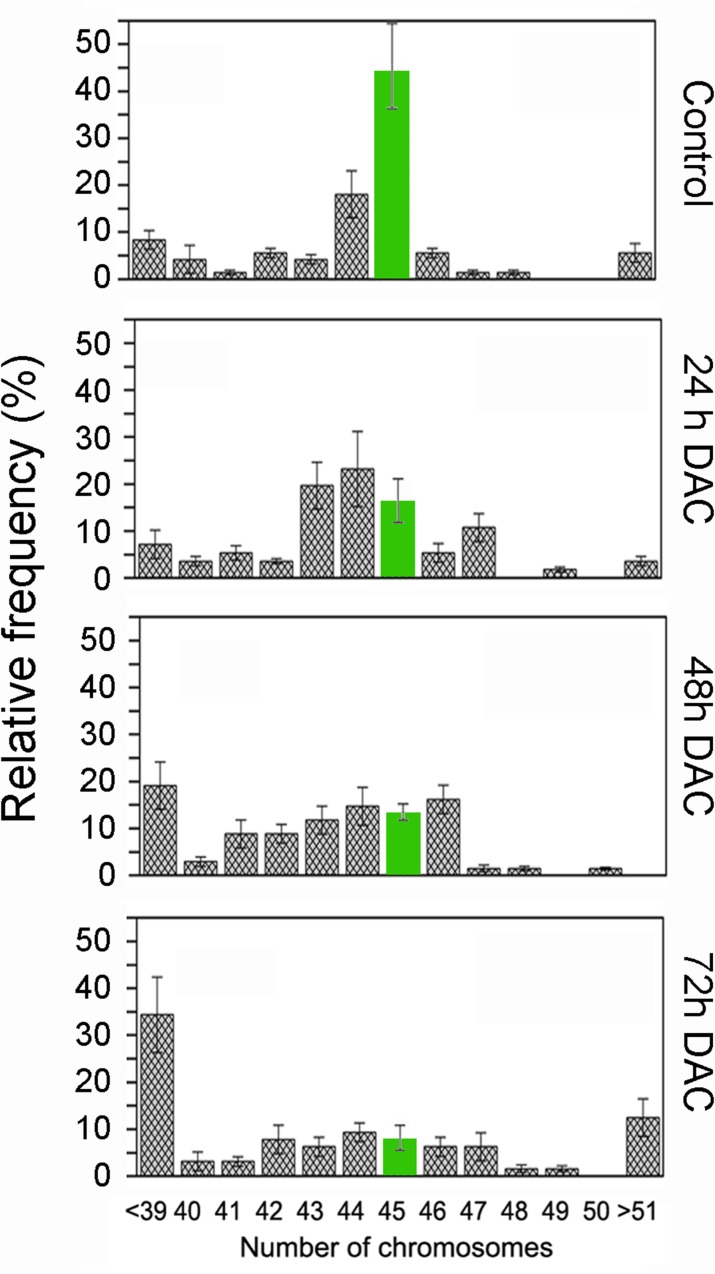
DAC induces aneuploidy in HCT-116 cells Histogram of Ploidy data of HCT-116 cells untreated or treated with 5μM DAC for 24 h (n=56), 48 h (n=68) and 72 h (n=64). The data are plotted as relative frequency of the number of chromosomes per cell and are expressed as a percentage. (n= Number of methaphases analysed per sample; data are from 3 independent experiments).

As loss of DNA methylation could interfere with chromosome dynamics, we evaluated if the global DNA hypomethylation observed upon the DAC treatment was associated with aneuploidy. We scored the number of chromosomes/cell by fluorescent microscopy in HCT-116 cells treated for 72 hours with 5μM DAC. This analysis showed a significant alteration in the distribution of the number of chromosomes per cell after treatment with DAC when compared to the control. Specifically, after 24 hours of treatment we observed a 30% reduction in the number of cells with 45 chromosomes and concurrently a significant increase in aneuploid cells with a clear trend to hypodiploidy. Additionally, it was also observed a significant increase of hypodiploid cells (<39 chromosomes per cell) at 48 and 72 hours, ranging from 15% to 25 % respectively (Figure 3).

### DAC treatment induces abnormal chromosomal DNA methylation pattern

**Figure 4:**
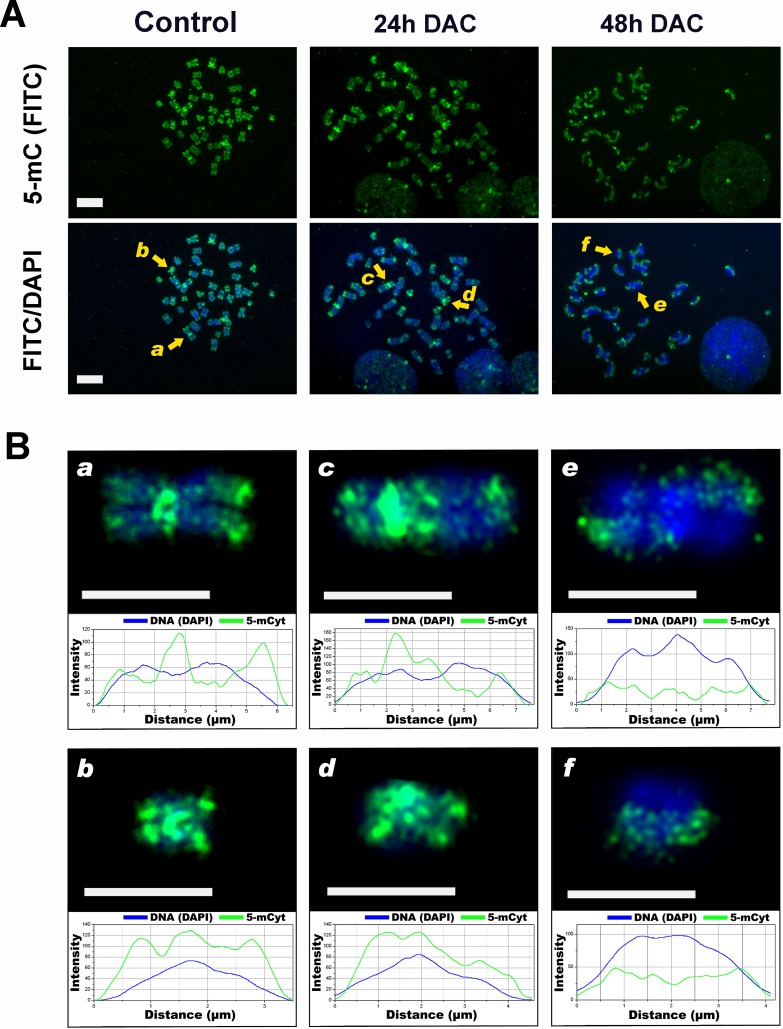
DAC alters the DNA methylation pattern of chromosomes A, immunofluorescence analysis shows the distribution of 5-mC on mitotic chromosomes. HCT-116 cells untreated or DAC-treated for 24 or 48h were stained using an anti- 5-mC monoclonal antibody conjugated to FITC (green signal), chromosomes were stained with DAPI (blue). Scale bars represent 10 μm. **B**. Magnified view of the chromosomes marked in **A**. with a yellow arrow. These chromosomes were chosen to perform a plot profiler analysis: chromosomes *a* and *b* for the control sample, *c* and *d* for the 24 h treatment sample and *e* and *f* for 48h treatment. The graphs were realised plotting the fluorescent intensity values (y-axis, arbitrary units) against the length of the scan (−axis) and show the DAPI profile (blue curve) and the distribution of methylation (green curve). Scale bar: 5 μm.

Recently, it has been proposed a relationship between loss of DNA methylation and chromosome segregation defects through the disruption of pericentromeric heterochromatin configuration [20, 23] [24, 25]. Given the increase in the number of aneuploid cells upon DAC treatment, we aimed to evaluate the changes in the methylation pattern of chromosomes by immunofluorescence microscopy to establish whether these changes could account for the mitotic segregation defects described. After 24 hours of DAC treatment, we did not observe a different 5-mC-FITC labelling of chromosomes in respect to untreated cells. However, after 48 hours chromosomes appeared asymmetrically labelled with one chromatid of each chromosome unlabelled (Figure 4). Moreover, pericentromeric regions appeared faintly labelled or not labelled at all (Figure 4B, e). The asymmetric methylation of sister chromatids is in agreement with data shown in Figure 2A and is indicative of loss of DNA methylation occurring after two subsequent cell cycles in presence of DAC. To relate the observed chromosome methylation pattern to the number of cell divisions that HCT116 cells underwent in the presence of DAC we determined cell proliferation timing by allowing cells to incorporate the halogenated thymidine analog 5-bromodeoxyuridine (BrdU) into DNA. Subsequently, by bivariate DNA-BrdU (propidium iodide (PI) and fluorescein isothiocyanate (FITC)) flow cytometry we analysed the presence of the BrdU-labeled cells during the cell cycle. The nuclei stained with PI, which fluoresces red at an intensity reflecting the DNA content, define a reference for G1 and G2/M cells. The cytofluorimetric profiles of HCT16 cells treated with DAC or left untreated were similar and suggest that HCT116 DAC treated cells duplicate in about 24 hours (Figure S2).

We gained additional evidence that DAC treated HCT116 cells have undergone two cell cycles using the Fluorescence plus Giemsa (FPG) technique to stain chromosomes. The FPG method is generally used to visualize sister chromatid exchanges in cells grown for two cell cycles in BrdUrd [30]. Based on semiconservative DNA replication, both chromatids of a chromosome will have incorporated BrdU into either one or both of their strands at the end of two cycles. After staining with the DNA-binding fluorochrome Hoechst 33258, UV photolysis, and Giemsa staining, the doubly substituted chromatids (new strands) appear light and the sister chromatids (parental strands) appear dark when observed by transmitted light microscopy (Figure S2B).

All together these findings confirm that the presence of asymmetrically methylated sister chromatids (Figure 4) is explained by the semiconservative DNA replication coupled with the passive loss of CpG dinucleotide methylation caused by DAC exposure (48h).

**Figure 5:**
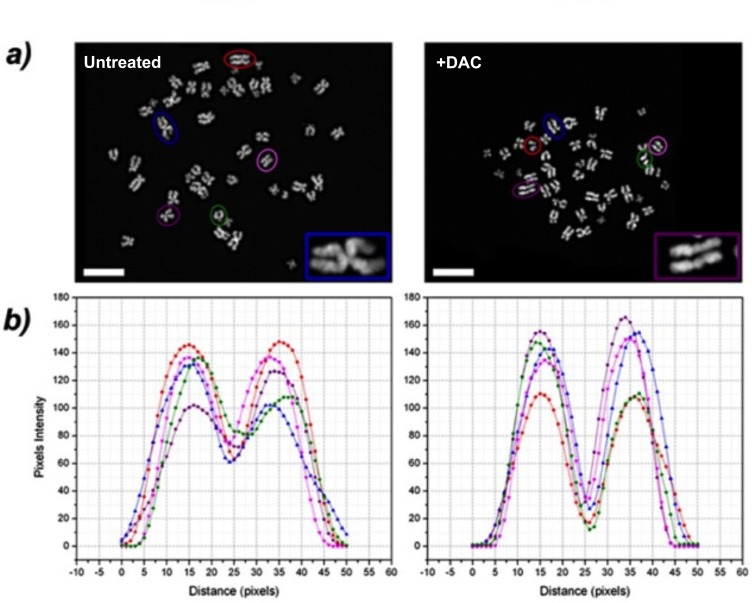
DAC induces “railroad track” chromosomes (RR) **A**. representative images of metaphases of untreated or DAC-treated HCT-116 cells. RR chromosomes were observed and a magnification is shown in the inset. Cells were stained with DAPI (in the inset is shown a magnification). Scale bars represent20 μm. **B**. DAPI signal intensity profile. The interchromatidic distance at the centromere was measured and the graph was realised plotting the distance in pixels of chromosome long axis in the x-axis and the pixel intensities along the chromosome short axis in the y-axis. The chromosomes analysed were selected from the metaphases shown in (A) (coloured circles) and are indicated in the plot by different coloured lines; y-axis:

In the attempt to understand how the induced DNA hypomethylation triggered aneuploidy, we looked at the morphology of chromosomes. In addition to chromosomal aberrations such as chromatid breaks/gaps, chromatid exchanges and chromosome pulverization (Figures S3, S4), we found “railroad track” chromosomes (RR) characterised by the absence of a clear centromere in 15% of mitosis analised. RR chromosomes coexisted with normal chromosomes in the same metaphase spread (Figure 5). Chromosomes in mitosis were DAPI stained to visualize the lack of a centromere with a plot-profiler method (Figure 5B).

### Extended DAC treatment causes aneuploidy and chromosome abnormalities

**Figure 6:**
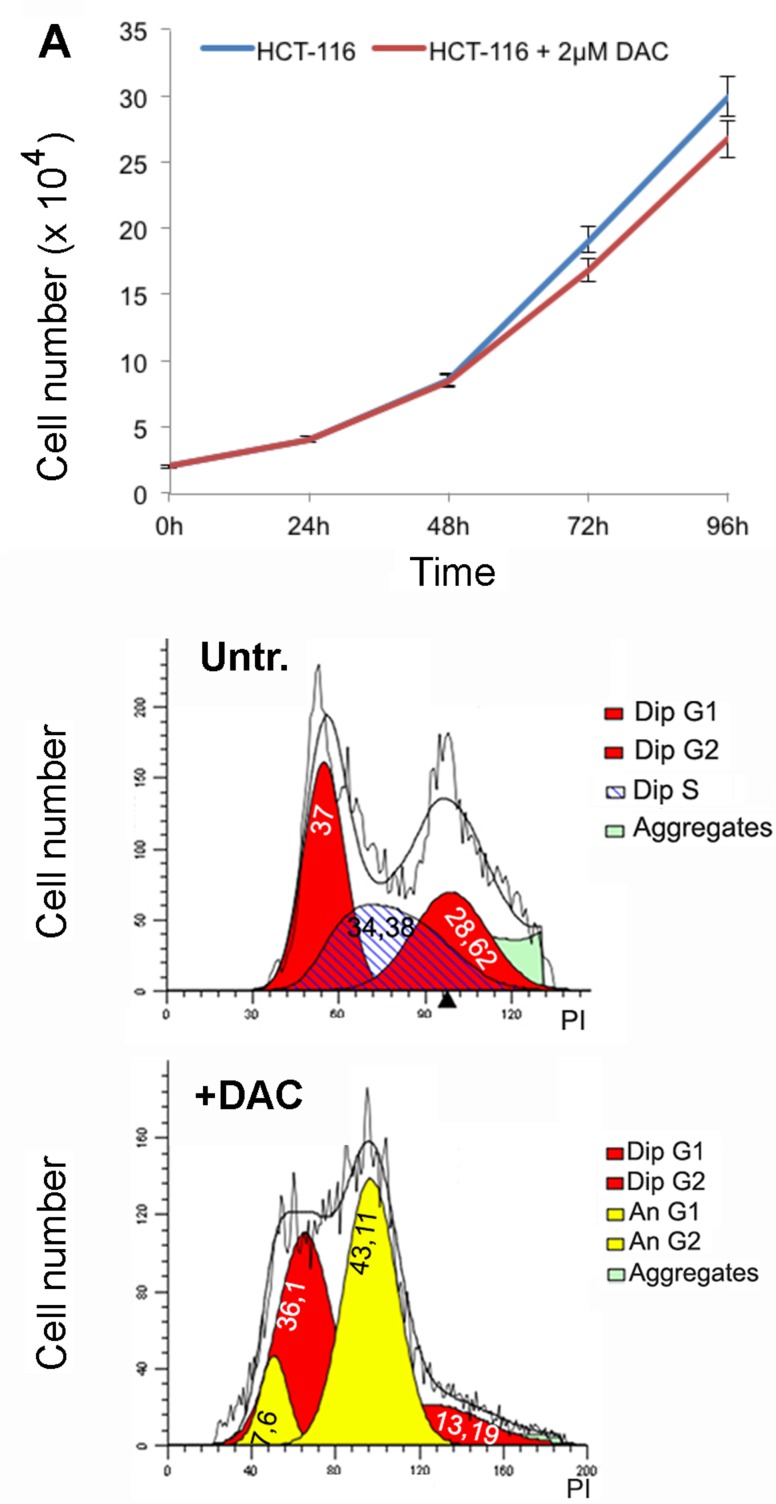
Extended treatment with DAC leads to aneuploidy **A**. Proliferation rates of HCT-116 cells treated with 2uM DAC **B**., **C**. Flow cytometry analysis shows cell cycle distribution of HCT-116 cells untreated or treated for one week with 2μM DAC. The flow cytometry data were analysed using the ModFit software.

We addressed then the question if an extended DAC treatment, up to 2 weeks, at lower doses than 5μM could have a different impact on cell viability and ploidy. Cells were then treated with 2μM DAC and counted every 24 hours to estimate the proliferation rate that resulted slightly delayed in respect to untreated cells (Figure 6A). In addition, the cellular ploidy was assessed after one week of treatment by using the ‘modfit’ software that estimates the ploidy of the cells after FACS analysis by measuring the DNA content (Propidium Iodide fluorescence). The results indicated the presence of an elevated percentage of aneuploid cells (50%) in these treated cells (Figure 6C).

**Figure 7:**
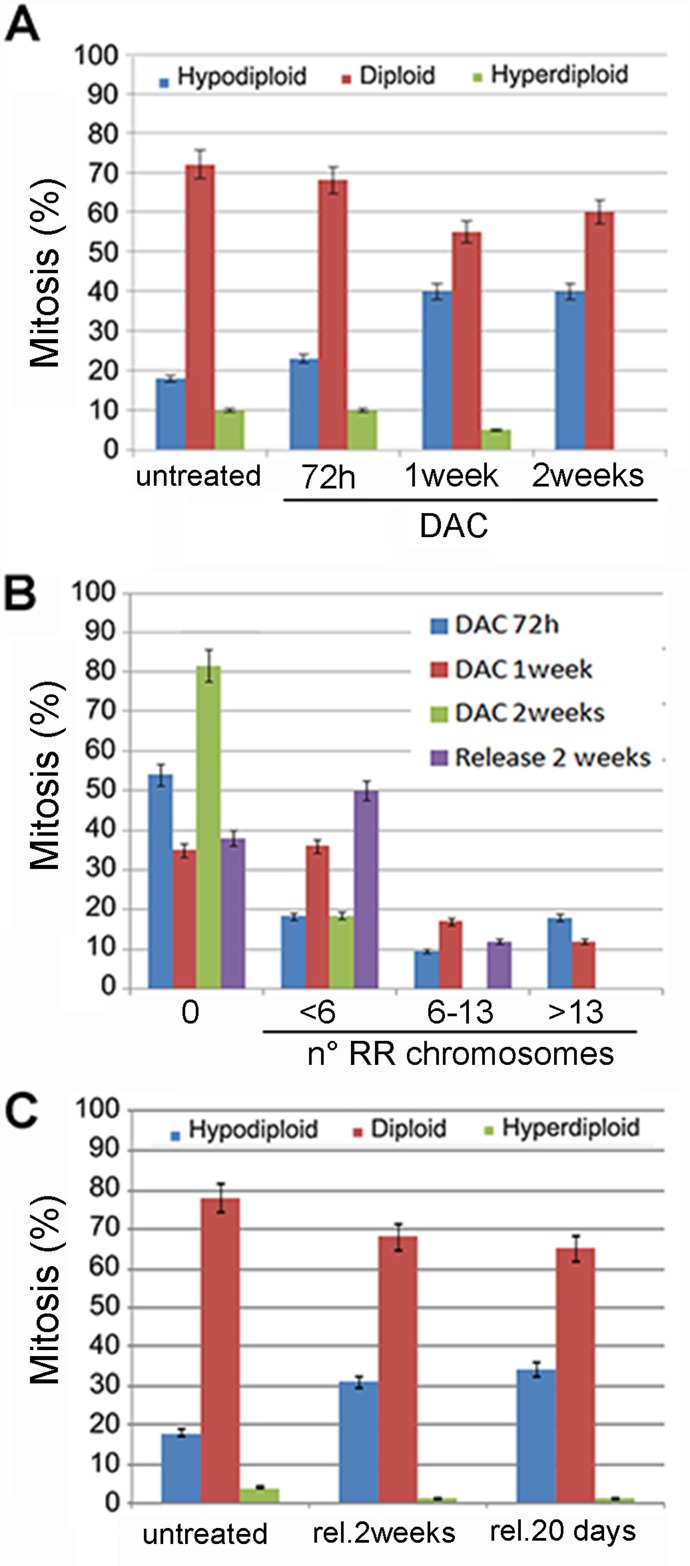
Prolonged DAC treatment causes aneuploidy and railroad track chromosomes **A**. The histogram relative to the ploidy of HCT-116 cells untreated or treated with 2μM DAC for the indicated period of time is shown in. **B**. the histogram shows the percentage of HCT-116 cells with railroad track chromosomes (x axis). Cells were treated with 2μM DAC for one and two weeks and released for two additional weeks. **C**. the histogram shows the percentage of aneuploid and diploid metaphases of HCT-116 cells treated with 2μM DAC and released (rel.) for the indicated times.

We determined then the effects of 2μM DAC treatment on aneuploidy generation at different times by evaluating chromosome numbers at 72 hours, 1 week, 10 days and 2 weeks from the beginning of the treatment. Seventy-two hours post-treatment cells showed aneuploidy and in particular 23% of aneuploidy cells were hypodiploid in which only few chromosomes were missing. This percentage increased up to 40% and remained stable up to two 2 weeks of treatment (Figure 7A). The prolonged treatment with 2μM of DAC also induced RR chromosomes (Figure 7B; Figure S5). These aberrant chromosomes were mainly evident after one week of treatment when the about 70% of metaphases showed at least a couple of RR chromosomes in which the centromere appeared less Giemsa stained. Taken together these experiments showed that DNA hypomethylation caused by DAC treatments induced in addition to aneuploidy, alteration of chromosome methylation pattern and of cell division. To establish whether the effects of the DAC treatment were reversible, cells exposed to 2μM DAC for one week were released in DAC-free medium for additional 14-20 days. After the release from the drug we observed a decrease in the number of aneuploid cells as well as of RR chromosomes suggesting that the effect of the DAC were partially reversible (Figure 7C).

### Aneuploidy triggered by hypomethylation is associated with mitotic defects

**Figure 8:**
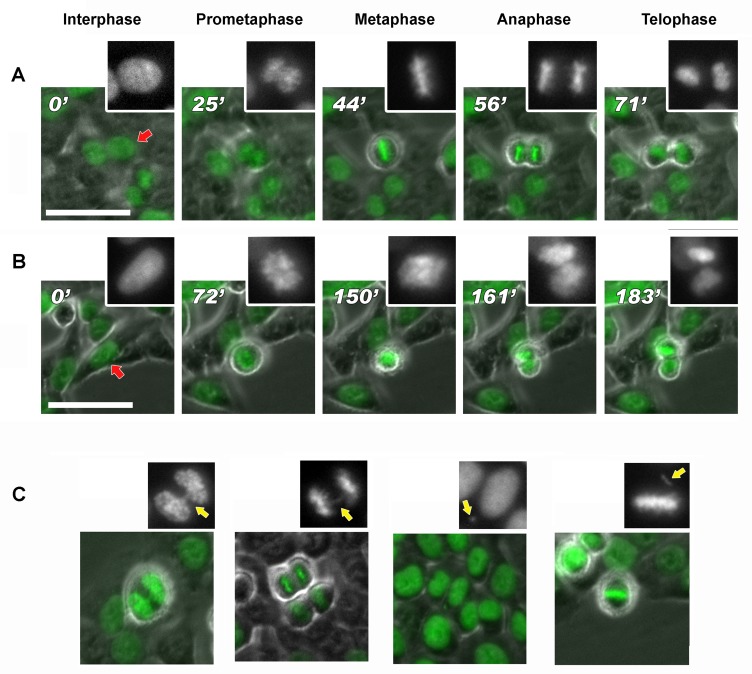
DAC induces mitotic delay and chromosomal defects Frame collection of DAC-treated cells progressing in mitosis obtained by time-lapse imaging. The DAC treatment (**B**) results in a delay of the anaphase onset for more than 40 min compared to the untreated control (normal mitosis in A). Images were acquired with phase contrast and GFP fluorescent filter. Scale bar represent 50μm.

We investigated if the altered methylation pattern of chromosomes correlated with chromosomes segregation defects. To this purpose, we performed live-imaging of DAC-treated HCT-116 cells expressing H2B-GFP [31] to detect mitotic abnormalities from which aneuploidy could arise. At 24 and 48 hours after DAC treatment (5μM), we observed increased mitotic defects and chromosome alterations like: chromosome bridge, lagging chromosome, micronuclei and chromosome out of the spindle. (Figure 8 and S4, Table 1). We noticed a strong increase (>40%) of these mitotic alterations at 48 hours from DAC treatment in agreement with the observed aneuploidy (Figure 3). The delay from prometaphase to anaphase progression was likely caused by the presence of these defects (Figure 8B).

**Table 1:** Quantification of segregation defects

HCT116	LC	MN
*Ctrl*	11%	2,5%
*+DAC 24h*	24,5%	11%
*+DAC 48h*	38%	6%

In the table are summarized the percentages of Lagging Chromosomes (LC) and Micronuclei (MN) observed durng time lapse analysis (Figure 8).

## DISCUSSION

Epigenetic marks combine to bring about DNA hypercondensation typical of centromeric and pericentromeric heterochromatin [32, 20, 22]. The maintenance of the epigenetic memory at these regions is believed to be essential for a functional centromere. Thus, loss of DNA methylation altering this configuration can result in kinetochore dysfunction so losing the connection between chromosomes and the mitotic spindle an event known to cause chromosome missegregation and aneuploidy [33]. Imbalance in cytosine methylation (hypomethylation) and deregulation of DNA methyl-transferases, are recurrent in human sporadic cancers and it was reported that they could be involved in the acquisition of chromosomal instability [34]. In a recent work, we demonstrated that DNA hypomethylation caused by DNMT1 post-transcriptional silencing induced aneuploidy in cells lacking p14ARF [14]. Here, we treated cells with low doses of DAC (2μM and 5 μM) in order to induce DNA hypomethylation without interfering with cell viability and we determined that DAC induces loss of DNA methylation at the centromere/pericentromere region resulting in mitotic defects (i.e. lagging chromosomes and micronuclei) that in turn underlie aneuploidy as suggested by the increase number of aneuploidy cells that raised up to 25-30% after DAC treatment. DAC acts through the formation of covalent adducts DNMT1-DAC-DNA leading to two consequences: the decrease of DNMT1 into the cell and CpG hypomethylation [28]. Cells exposed to this drug for either a short (72 hours) or a long (1-2 weeks) period of time became aneuploid. This condition was alleviated when cells were released in drug-free medium for additional two weeks. The decrease in DNA methylation was assessed by slot blot assay that evaluates the global degree of DNA methylation and by Methylation Specific PCR. Both strategies confirmed that low doses of DAC induce not only a global reduction in 5mC levels, but also the specific methylation loss of the CHFR gene promoter. Interestingly, the reduction in global DNA methylation was associated with a significant change of the 5mC pattern along mitotic chromosomes in that the presence of 5mC was detected in only one of the two sister chromatids after 48 hours of DAC treatment. This finding is in agreement with the semiconservative replication of the DNA in that after two cell divisions, one chromatid is originated by the unmethylated DNA strands resulting from the passive loss of DNA methylation induced by DAC. The other chromatid, instead, retains the original methylated strand. Our results also confirmed the HCT116 cell cycle duration of about 24 hours.

We hypothesize that DNA hypomethylation is directly responsible for the aneuploidy we observed. Our working model is supported by our time-lapse video-microscopy where we observed several mitotic alterations: mitotic delay, lagging chromosomes and micronuclei generation, all of them recognized as a cause of aneuploidy.

Moreover, we observed the RR chromosomes that are characterized by the lack of the centromere and appear as two single chromatids close to each other. These RR chromosomes are not a consequence of premature separation of the sister chromatids since not all the chromatids in a metaphase appeared separated. Instead they appear to be the consequence of the loss of DNA methylation at the pericentromeric region of these chromosomes that affects the centromere organization.

Although we cannot exclude that DNA hypomethylation might also affect genes involved in chromosome dynamics leading to destabilization of chromosome cohesion and segregation, our results suggest that the altered DNA methylation pattern could compromise the functionality of the centromere and consequently the correct chromosome segregation.

Finally, our results point at DNA hypomethylation as a novel mechanism underlying aneuploidy and further studies should be aimed to evaluate the integrity and functionality of centromeres and kinetochores in cells undergoing DNA methylation loss.

## MATERIALS AND METHODS

### Cell culture

Human colon cancer HCT-116 cells, characterized by a chromosome number of 45, were cultured in DMEM medium supplemented with 10% foetal calf serum, 100 U/ml penicillin, 100 μg/ml Streptomycin and 1 mM Sodium Pyruvate (all from Gibco, Life Technology, Italy). Cells were grown at 37°C in a 4% CO_2_ humidified atmosphere and treated with 1, 2 and 5 μM of DAC (SIGMA-Aldrich, Italy) 24h after plating. Fresh medium supplemented with DAC was replaced every 24/36h. Assays were conducted on extracts harvested 1, 2 and 3 days or 1 week after the beginning of DAC treatment.

### Evaluation of cell viability by the MTT assay

Cell viability was assayed by using [3-(4,5-dimethylthiazole-2-yl)-2,5-diphenyltetrazolium bromide] (MTT, SIGMA-Aldrich, Italy). Cells were seeded in a 96-well plate at a density of 10^3^ cells/well in 200μl of culture medium with only vehicle or DAC at different concentrations. Cells were incubated at 37°C in a humidified incubator at 5% CO_2_. Additionally, six wells were left empty for cell-free background reference. The medium was refreshed with a solution containing 180μl medium plus 20μl of 5mg/ml MTT stock solution in PBS to each well (0.5 mg/ml final concentration). The microplate was incubated for 4h at 37°C, 5% CO_2_. The plate was centrifuged at 1000 rpm for 10 min and the medium totally removed from each wells followed by the addition of 100μl of DMSO to solubilise the MTT formazan. The absorbance of each well was measured at 570nm on a multiplate reader (GloMax®-Multi Detection System, Promega, Italy) and the data saved as text file and imported into the statistical software Origin-Pro 8.5.1. (OriginLab Corporation) for processing. The percentage of viable cells was measured as follows:

$$\%\text{ viability }=\;\frac{\text{optical density of cells treated with drugs}}{\text{optical density of cells treated with only vehicle}}$$

### Cell viability by Acridine Orange/Ethidium Bromide (AO/EB) Staining

A mixture of Acridine Orange was used for live cell indentification and Ethidium Bromide for discrimination between live and dead mammalian cells on the basis of membrane integrity. Cells were seeded in a 96-well plate at a density of 10^3^ cells/well in 200μl of culture medium with only vehicle or DAC at different concentrations and times and incubated at 37°C, in a humidified incubator at 5% CO_2_. For the analysis, the medium was removed and 20μl of the Acridine Orange (100μg/ml), Ethidium Bromide (100μg/ml) mixture (1:1, v/v) was added into each well and incubated for 20 seconds. The mixture was then removed and cells observed with fluorescent microscopy

### Cytofluorimetric analysis

HCT-116 cells were grown into complete medium (DMEM) and treated with DAC up to 48 hours. The DNA content was determined staining cells with 4μg/ml of Propidium Iodide (PI, SIGMA-Aldrich, Italy). The PI solution was prepared in PBS and it was supplemented with 40μg/ml RNase. Analysis of PI labelled cells was conducted as described previously [14] and samples were analysed on a FACSCanto (Becton Dickinson).

Bivariate analysis to evaluate cell cycle progression/duration where done by pulse labelling (1 h) cells with bromodeoxyuridine (BrdU 10μM). Analysis of BrdU labelled cells was conducted as described previously [14] [35]; briefly cells were fixed and stained with anti-BrdUFITC antibody (biolegend USA), to detect BrdU positive cells (S-phase) and propidium iodide (PI) to assess DNA content and samples were analyzed on a FACSCanto (Becton Dickinson). Experiments were repeated at least twice and 10,000 events were analysed by using the softwares: FACSDiva (Becton Dickinson) and ModFit (Verity Software House, Inc.).

### Cytogenetics analysis

Cells were treated with 0.2μg/mL of colcemid (Demecolcine, SIGMA-Aldrich, Italy) for two hours, trypsinized and harvested by centrifugation at 1000 rpm for 10 min. Cells were swollen by adding 75mM KCl dropwise and incubated at 37°C for 10 min, then centrifugated at 800 rpm for 10 min. The pellet was resuspended adding dropwise 5 mL of cold Carnoy's fixative [methanol/acetic acid (3:1 v/v)] and incubated for 15 min on ice. After repeating the last step twice, cells were dropped onto iced slides. Chromosomes were stained either with Giemsa or with 1μg/mL of DAPI (4′,6-diamidino-2-phenylindole, SIGMA-Aldrich, Italy) and examined on a Zeiss Axioskop microscope equipped for fluorescence, images were captured with a CCD digital camera (AxioCam, Zeiss) and then transferred to Adobe PhotoShop. We evaluated at least 100 mitoses for each sample. The experiment was repeated twice.

### Fluorescence plus giemsa differential (FPG) chromosome staining

Asynchronous HCT116 cells to which BrdUrd (SIGMA-Aldrich, Italy) was added for 48 h were mitotic arrested with 0.2μg/ml of Demecolcine (SIGMA-Aldrich, Italy) for 1 h. Differential staining was conducted as previously described [30]; briefly, fixed slides were stained with Hoechst 33258 (0. 1 μg/ml) in PBS, placed under an UV lamp for 25 minutes, incubated in 2X SSC for 15 minutes at 65°C and then stained with 3% Giemsa in a phosphate buffer (PBS) for 10 to 20 minutes. Picture of Differentially stained metaphases were saved in Adobe Photoshop.

### Evaluation of 5mC content by immunofluorescence microscopy

DNA of fixed cells was denatured in 70% formamide/2X SSC (v/v) at 70°C for 5 min. The slides were then dehydrated in a series of 70, 80 and 100% cold ethanol and air dried. Samples were incubated in a blocking solution (3% bovine serum albumin, 0.1% Tween-20/PBS) in coplin-jar for 1 hour. An anti- 5-methylcytosine (5-mC) mouse monoclonal antibody (cloneD33, Epigentek, USA) was diluted 1:100 in 1% Tween-20/PBS,) and was added to the cells for 1 hour at 37°C in a humidified chamber. The slides were washed with 0.05% Tween-20/PBS, 3 times for 10 min and incubated with FITC conjugated anti-mouse secondary antibody (diluted 1:1000 in 1% Tween-20/PBS) for 1 hour at 37°C in a humidified chamber. Finally, cells were washed with 0.05% Tween-20/PBS 3 times 10 min each and nuclei were stained with 1μg/mL of DAPI. Fluorescent images of prophase nuclei were acquired using the Zeiss Axioskop microscope equipped for fluorescence. To estimate the fluorescent signal of each nucleus images were processed using the ImageJ software. The intensity of the 5-mC signal was calculated as integrated density and processed using the statistical software OriginPro 8.5.1 (OriginLab Corporation) as corrected total cell fluorescence (CTCF) according to the following formula: CTCF = Integrated Density − (Area of selected cell × Mean fluorescence of background readings). The final value was normalized with DAPI signal. The same slides were used to evaluate the 5mC methylation pattern of metaphase chromosomes. For each condition tested, chromosomes of 50 metaphases were scanned along their longitudinal axis by using the RGB profile plot plugin of ImageJ that provided, for every rectangular selection, a two-dimensional graph of the intensities of pixels along a line and a plot list of the data, in which were reported the values of pixel intensity for each channel (blue, DAPI; green, 5-mC signal). That data were then processed with Origin-Pro 8.5.1 (OriginLab corporation) and graphed in two different colour lines for each fluorescent channel, where the x-axis represents the horizontal distance in microns through the chromosome and the y-axis the vertically averaged pixel intensity.

### Slot blot analysis

The DNA-slot blot analysis of 5-mC was performed as described in [36] with the following modifications. Non-methylated DNA of Escherichia coli ET12567/pUZ8002 [37] was used as negative control and methylated DNA of Escherichia coli Yale BW25113 [38] as positive control. Total genomic DNA was purified and 100 μg/25 μL from each sample were denatured at 100°C for 5 min and applied onto Hybond-N membrane (RPN203N, Amersham Biosciences) using the Hybrislot Manifold Apparatus (Whatman Biometra, Germany) under vacuum for 3 min. DNA was fixed to the membrane by exposing to UV light (70,000 μ joules/cm^2^) for 45 seconds using the Hoefer UVC 50 Crosslinker (Amersham Biosciences). The membrane was then incubated in 5% w/v no-fat dry milk prepared in TBST buffer (Tris Buffered Saline 0.05% Tween-20), for 1 hour at RT. This step was then followed by incubation with anti- 5-Methylcytosine Monoclonal Antibody (1:500, cloneD33, Epigentek, USA) for 1 hour at RT. The membrane was then washed 3 times with TBST for 10 min and incubated with an anti-mouse HRP conjugated secondary antibody diluted 1:2000 (Abcam, UK) for 1 hour at RT. After extensive washes in TBST (3 washes of 10 min each) the membrane was washed once in double distilled water and developed by enhanced chemiluminescence detection reagents (Pierce, Thermo Scientific). Images were acquired with the Chemidoc XSR Imaging System (BioRad). The amount of spotted DNA was detected by staining the membrane with 0.02% w/v methylene blue in 0.3 M sodium acetate (pH 5.2). Subsequently, washing in double distilled water was necessary to reduce the background noise. The relative 5-mC optical density (OD) value was calculated with Quantity One 4.6.7 software (BioRad) and the amount of DNA was normalised against the wild type control.

### Methylation specific PCR (MSP)

Genomic DNA was extracted from DAC treated HCT-116 cells (1, 2 and 5μM) by using the All-Prep DNA/RNA kit (Qiagen S.r.l.- Italy). 2μg of DNA in a volume of 20μl were converted by Epitect Bisulfite kit (Qiagen S.r.l.- Italy). Methylation-specific PCR amplification was carried out using oligonucleotide primers which were designed to anneal specifically to either methylated or unmethylated DNA after sodium bisulfite conversion as described above. The oligonucleotides used in this study were the following: Methylated DNA-specific primers were MCHFR-forward (5′-ATATAATATGGCGTCGATC) and MCHFR reverse (5′-TCAACTAATCCGCGAAACG). Unmethylated DNA-specific primers were UCHFR-forward (5′-ATATAATATGGTGTTGATT) and UCHFR reverse (5′-TCAACTAATCCACAAAACA) [39].

The PCR reaction was performed using 100ng of DNA and the following reagents mix: 1x Buffer, 0,2 μM dNTP mix, 1,5 mM MgCl_2_, 0,5 μM Primer for, 0,5 μM Primer rev, 1,5U/μl Taq (Applied Biosystems, Thermo Fisher Scientific) PCR reaction consisted of a step at 95°C for 10min before of 45 cycles at 94°C for 1 min, 58°C for 1min, 72°C for 1 min (MCHFR); 94°C for 1 min, 50°C for 1 min, 72°C for 1 min (UCHFR), followed by a final step at 72°C for 4 min. The resultant PCR products were resolved on a 1,8% agarose gel.

### Reverse transcription PCR (RT-PCR)

Primers to be used in real time RT-PCR experiments were designed with Primer Express software (Applied Biosystems, Life Technologies) choosing amplicons of approximately 70-100 bp. The selected sequences were tested against public databases (BLAST) to confirm the identity of the genes. Total RNA was extracted from cells by using the “PureLink RNA mini kit ” (Ambion, Thermo Fisher Scientific) according to the manufacture's instruction. RNA was reverse-transcribed in a final volume of 20μL using the High Capacity cDNA Reverse Transcription kit (Applied Biosystems, Thermo Fisher Scientific) for 10 minutes at 25°C and 2 hours at 37°C. For each sample 50ng of cDNA, was analyzed by RT-PCR (95°C for 15 sec, 60°C for 60 sec repeated for 40 cycles). RT-PCR was done in a final volume of 20μl comprising 1x Master Mix SYBR Green (Applied Biosystems, Thermo Fisher Scientific) and 0,3 μM of forward and reverse primers for: GAPDH (Fw: 5′-CTCATG ACCACAGTCCATGCC-3′, Rev: 5′-GCCATCCACA GTCTTCTGGGT-3′), CHFR (Fw: 5′-CCTCAACAACCTCGTGGAAGCATAC-3′, Rev: 5′-TCCTGGCATCCATACTTTGCACAT-3′).

### Western blotting

Protein concentration was measured using the Bio-RadProtein Assay (Bio-Rad Laboratories). Proteins (40 μg) were separated by 4-12% Bolt bis-tris plus gels (Invitrogen, Thermo Fisher Scientific) and transferred to Hybond-C nitrocellulose membranes (Amersham Life Science) by electroblotting. The membranes were sequentially incubated with primary antibodies against CHFR (goat, Santa Cruz, 1:100), β-tubulin (mouse, SIGMA, 1:5000) and HRP-conjugated mouse (Pierce Thermo Fisher Scientific, 1:5000) or goat (ab97110, Abcam, 1:2000) as secondary antibodies. The target protein was detected with enhanced chemiluminescence Western blotting detection reagents (Pierce, Thermo Fisher Scientific).

### Live imaging of cell division by time lapse video-microscopy

HCT-116 cells expressing H2B-GFP were seeded in 25 cm^2^ flasks and treated with DAC as reported above and cultured in 6 mL of complete DMEM medium (Gibco) 24 h before Time-Lapse analysis, the medium was replaced with supplemented HEPES no phenol red DMEM (Gibco, Life Technology, Italy). Fluorescent and phase contrast images were automatically acquired every 10-60 sec over a period of 1-4 h using the Epifluorescence inverted microscope Axio Observer D1 (Carl Zeiss). The microscope was controlled in an automated manner using the Axio-Vision40 V 4.8.2.0 software and housed in a home-made box to maintain a constant temperature of 37°C. Fluorescence and differential interference constrast images were obtained. When necessary, manual focusing was performed every 20 min using the fluorescence channel. Time-lapse images were processed in Adobe Photoshop CS5 indicating the movie progression time in minutes.

## SUPPLEMENTARY MATERIAL FIGURES


