# Supplementary material for: DNA demethylation caused by 5-Aza-2′-deoxycytidine induces mitotic alterations and aneuploidy
Source: Oncotarget. 2016 Jan 12;7(4):3726–39. doi: 10.18632/oncotarget.6897 (PMC4826165; doi:10.18632/oncotarget.6897)
Supplement: Supplementary file 1 [file oncotarget-07-3726-s001.pdf]

DNA demethylation caused By 5-Aza-2'-Deoxycytidine induces mitotic alterations and aneuploidy

Supplementary Material

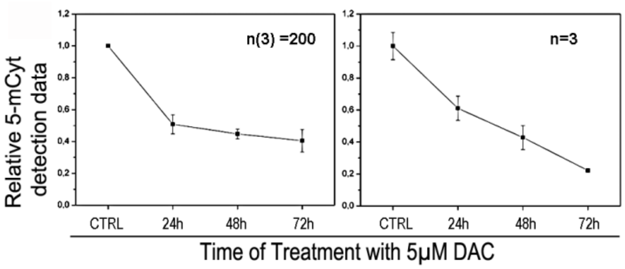

Figure S1. **Quantitation of the 5mC signal in Figure 2**. A, graph relative to 5-mC integrated density values is showed on the left. The graph was obtained by scoring 200 nuclei from 3 independent experiments. The graph on the right shows quantitation of 5-mC data relative to control sample from slot-blot analysis.

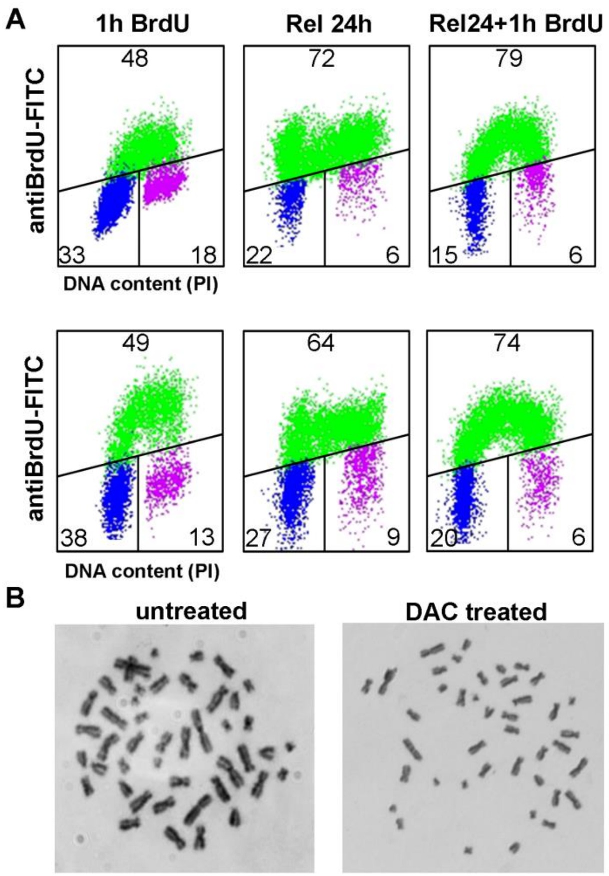

Figure S2. **Effects of DAC on the cell cycle duration of HCT116 cells**

HCT116 cells were fixed at the indicated times and analyzed using flow cytometry. *A,* representative Cytofluorimetric profiles (dot plots) of untreated and DAC-treated HCT116 cells pulsed with BrdU for 1 h and stained anti-BrdU antibody FITC-conjugated and propidium iodide (PI) The green dots indicate the BrdU positive cells that underwent DNA replication (S), blue and purple dots indicate cells with a 2N (G1) and 4N (G2/M) DNA content, respectively. *B,* representative differentially stained (FPG) HCT116 metaphases indicating that untreated and DAC treated HCT116 cells and chromosomes undergo two rounds of DNA replication during 48 hours of DAC exposure.

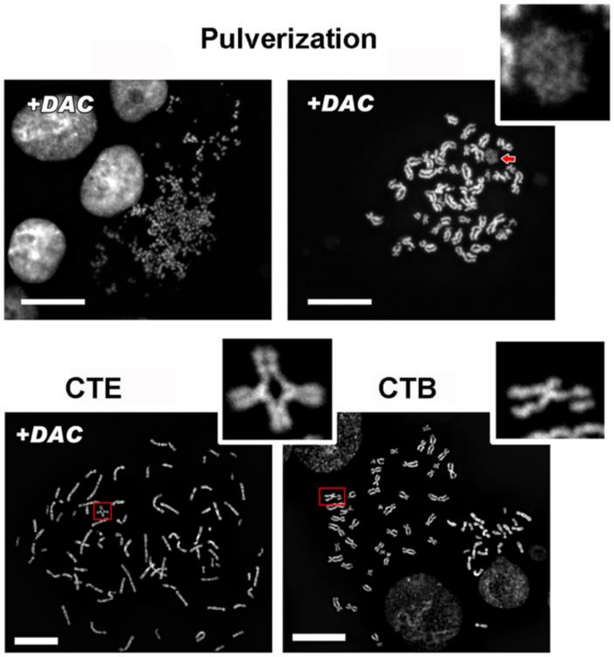

Figure S3. **Chromosome aberrations after DAC treatment**

Top panels: pulverization of all chromosomes (left) and of a single chromosome resembling chromotripsis. A magnification is shown in the inset. CTE, Chromatid Exchanges. CTB, Chromatid Breaks. Scale bar represent 20 µm. Chromosomes were stained with DAPI.

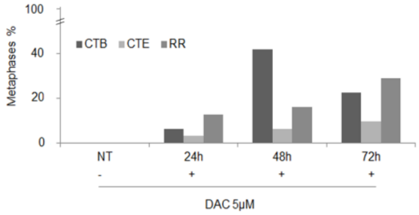

Figure S4. **Quantification of chromosomal defects after DAC treatment**

Histogram showing the quantification of chromosomal defects observed in figure 5 and figure 8 (RR: “railroad track” chromosomes, CTE: Chromatid Exchanges, CTB: Chromatid breaks).

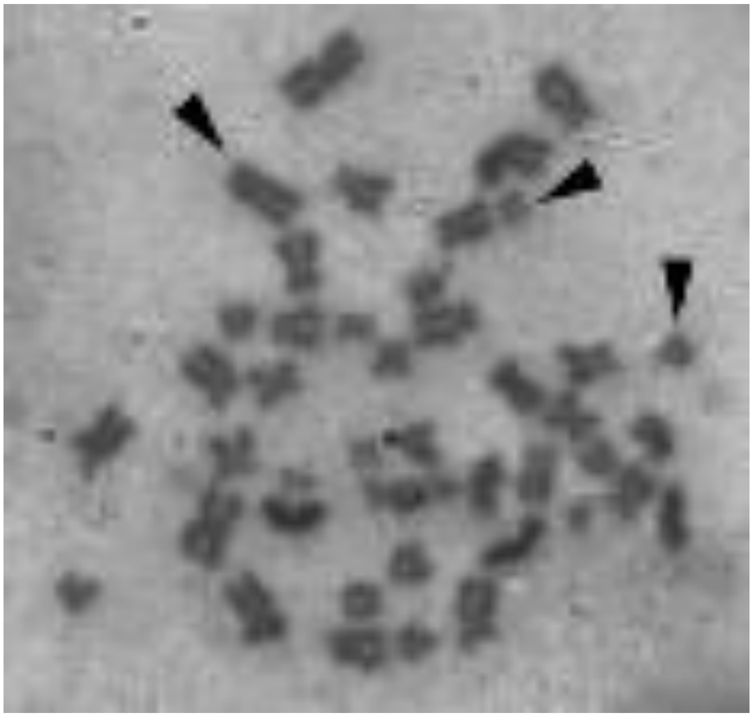

Figure S5. **Railroad track chromosomes after prolonged treatment with DAC**.

Representative picture of a metaphase showing the presence of railroad track chromosomes after one week of treatment with 2μM DAC. Arrows indicate ‘railroad track’ chromosomes.
